# Guidance Navigation and Control for Quadrotor UAV Using Lyapunov-Based Backstepping

**DOI:** 10.3390/s26092611

**Published:** 2026-04-23

**Authors:** Jurek Z. Sasiadek, Ammar Shuker, Malik M. A. Al-Isawi

**Affiliations:** Department of Mechanical and Aerospace Engineering, Carleton University, Ottawa, ON K1S5B6, Canada; jurek.sasiadek@carleton.ca (J.Z.S.); ammarshuker@cmail.carleton.ca (A.S.)

**Keywords:** quadrotor UAV, lyapunov stability, backstepping control, PSO, FOPID

## Abstract

Quadrotor UAVs present a significant control challenge due to their underactuated nature; strong coupling effects; nonlinear dynamics; and high sensitivity to unknown effect parameters, external disturbances, and uncertainties. To address this issue, this study proposes a Lyapunov-based backstepping (LYP) controller that ensures robust stability and precise trajectory tracking. The controller employs an inner- and outer-loop architecture for coupled position and attitude control. Its performance is compared with Proportional–Integral–Derivative (PID) and Fractional-Order PID (FOPID) controllers under three scenarios: nominal conditions, external disturbances, and model parameter uncertainties. All controller gains are optimized using Particle Swarm Optimization (PSO). Simulation results, which are evaluated using time-domain metrics and root mean square error (RMSE), demonstrate that the proposed LYP controller achieves superior robustness, faster disturbance rejection, and improved tracking accuracy compared to both PID and FOPID controllers.

## 1. Introduction

Due to the increasing significance of UAVs in both civilian and military applications, research on these systems has grown rapidly in recent years. UAVs have attracted considerable interest from scientists and designers due to their impressive advancements compared to other robotic platforms. Consequently, various control mechanisms have been set to improve their robustness, precision, and stability. A human pilot can operate a UAV autonomously or remotely. Among different UAV types, quadrotors are popular due to their mechanical simplicity, maneuverability, and ability to hover and perform precise maneuvers. Recent progress in information systems, sensing technologies, and computational hardware has further enhanced UAV capabilities. As a result, quadrotors are currently used in a wide range of applications, including defense operations, agricultural spraying, medical supply delivery, monitoring, surveying, mineral exploration, fighting fires, and rescue operations. These growing applications underscore the need for dependable, robust control systems that can ensure safe, effective operation in practical settings. Jia et al. [1] propose a unique nonlinear control approach and simulate it for a UAV helicopter. A nonlinear control approach employing integrated backstepping and sliding-mode control was used to achieve accurate trajectory tracking and control the UAV’s attitude. A novel hybrid adaptive nonlinear control technique for quadrotor UAVs is presented by the authors of [2]. To deal with the trajectory-following problem, a combined backstepping and sliding-mode control technique is developed based on improved PID sliding. Then, the Lyapunov stability method is used to analyze and ensure the stability of a closed-loop system. Next, an adaptive mechanism is built into the sliding-mode controller to estimate uncertain system parameters. Finally, a Super-Twisting algorithm is used to eliminate the chattering effects inherent in standard sliding-mode control. The proposed technique improves the robustness and accuracy of trajectory tracking in the presence of system uncertainties and external disruptions. An efficient adaptive sliding-mode controller for addressing the drone’s trajectory tracking problem in the presence of parameter-model uncertainty is presented in [3]. The PSO approach is used to optimize the controller variables, thereby improving tracking performance and resilience. It used PSO to utilize and optimize control variables for quadrotor attitude and tracking control. This technique combines a feed-forward path-following approach and a PD controller, with PSO used to optimize controller gains. The performance of the suggested approach is assessed using the RMSE as a validation parameter [4]. Nettari et al. [5] provided a direct optimum control strategy for a nonlinear system with multiple inputs and multiple outputs (MIMOs). The research focuses on developing a suitable model-free backstepping controller for a MIMO quadrotor subject to uncertain external disturbances. The suggested method leverages a model-free backstepping control framework with parameters optimized via a cuckoo search algorithm. A robust adaptive tracking control approach for quadrotor systems with unknown characteristics, such as thrust and drag coefficients, is described in [6]. To address the issues from uncertain input gains, an adaptive linearizing control strategy is used, and it is implemented consistently in both the outer-loop and inner-loop control designs to ensure correct tracking performance. The authors presented a new resilient fault-tolerant control method using backstepping, which was created, developed, and experimentally tested on a quadrotor testbed for tracking control while operating under an actuator fault situation. Backstepping is well known for its ability to maintain system performance while reducing sensitivity to disturbances. Building on this feature, the proposed nonlinear controller employs an enhanced robust backstepping strategy to efficiently address system uncertainties from both external disturbances and actuator failures. Ullah et al. [7] presented a new resilient finite-time control approach for a class of underactuated nonlinear systems. To ensure finite-time stability at the intended equilibrium points, the proposed method combines an integral terminal fractional-order sliding-mode controller with a reaching-phase-free integral backstepping algorithm. Li et al. [8] outline a unique hybrid control technique for quadrotor UAVs based on neural dynamics. This method effectively addresses two fundamental drawbacks of existing approaches: velocity discontinuities arising from classical backstepping control and control-signal chattering from traditional sliding-mode control. A prescribed-time observer-based sliding mode controlling law for nonlinear systems with disturbances and inner parameter dynamics is presented in [9]. This method is employed with a prescribed-time extended-state observer for a common flying control task: ensuring the roll stability of a precisely guided aircraft vehicle.

A backstepping sliding-mode technique is used to design an adaptive and robust tracking-trajectory controller for a quadrotor operating under unpredictable conditions. The controller uses adaptive estimation to estimate unknown system model parameters and includes an interference-prevention mechanism to mitigate the impact of external disturbances. This technique compensates for rotor control inputs, improving the precision and stability associated with path control [10]. Ranjan and Majhi [11] address the orientation stability issue faced by quadrotors when dealing with unknown inertia, external perturbations, and actuation defects within a predetermined time period. To overcome these issues, they use an adaptive, predefined-time sliding-mode controller with a radial basis function neural network. This strategy maintains the target trajectory while effectively assessing system uncertainties, thereby improving control performance. The main objectives are the development of an advanced control system for robust, stable and accurate guidance and navigation. This paper presents a new Lyapunov-based backstepping (LYP) controller for improving system performance. The controller maintains consistent, stable operation in the presence of external disturbances. It also effectively addresses parameter uncertainties that influence system dynamics. PSO is used to optimize controller gains while maintaining Lyapunov stability.

The key innovations of this work include:

(i) The development of a Lyapunov-based backstepping controller with enhanced robustness for nonlinear quadrotor dynamics.
(ii) The integration of PSO for systematic and optimal tuning of controller gains.
(iii) A comprehensive comparative evaluation against PID and FOPID controllers under multiple challenging scenarios.

The remainder of this paper is organized as follows: Section 2 depicts the mathematical model of quadrotor UAV. Section 3 presents the Fractional-Order PID controller. Section 4 describes the optimal FOPID controller using PSO. Section 5 details the proposed LYP controller. Section 6 explains the optimal gains of Lyapunov-based control. Section 7 discusses the results and analysis, and finally, Section 8 presents the conclusion.

## 2. Mathematical Model

**Figure 1:**
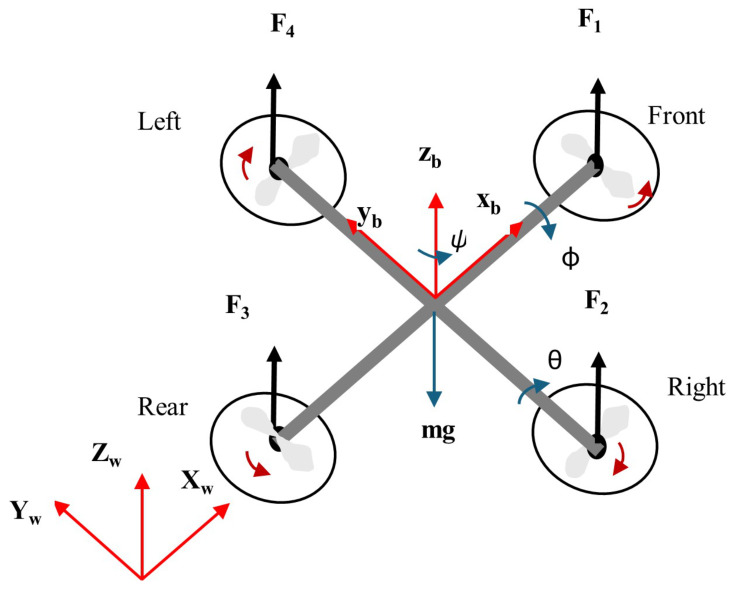
The quadrotor structure.

This section discusses the mathematical model of the quadrotor. As seen in Figure 1, the quadrotor in this study contains four motors with different angular velocities to produce forces. As a result, the Newton–Euler equation governs the kinematic and dynamic models. The vertical movement in a quadrotor design results in an increase or reduction in the speed of each rotor. Pitch movement is produced by altering the first and third rotor speeds in opposition to the roll motion caused by altering the second and fourth rotor speeds. Yaw motion is produced by the torque differential between two rotors that rotate clockwise and two rotors that rotate counterclockwise. With only four inputs to control and six degrees of freedom (position and attitude), the quadrotor system is typically underactuated and nonlinear [12]. The quadrotor body frame ($x_{b},\;y_{b}$, and $z_{b}$) is defined in the ENU coordinate system, as illustrated in Figure 1. The frame is fixed such that the $x_{b}$ axis points east, the $y_{b}$ axis points north, and the $z_{b}$ axis points upward. The body frame and the world frame ($X_{w},\;Y_{w}$, and $Z_{w}$) are locked to the same origin.

### 2.1. Kinematic Model

The rigid-body dynamics are formulated into the translational and rotational dynamics of the quadrotor. Therefore, the translation position vector is $\xi ={\left[x,\;y,\;z\right]}^{T}$, and the attitude angle vector is represented as $\zeta ={\left[\phi ,\;\theta ,\;\psi \right]}^{T}$, where the roll and pitch angles are bound between −π/2 and π/2, while the yaw angle is bound between −π and π. The rotation matrix from the body frame to the world frame is presented by the *R* matrix [13].

(1) $$R=\left[\begin{array}{ccc} c_{\theta }c_{\phi } & -c_{\psi }s_{\phi }+s_{\theta }c_{\phi }s_{\psi } & s_{\phi }s_{\psi }+c_{\phi }s_{\theta }c_{\psi }\\ c_{\theta }s_{\phi } & c_{\psi }c_{\phi }+s_{\theta }s_{\phi }s_{\psi } & -c_{\psi }s_{\phi }+c_{\phi }s_{\theta }s_{\psi }\\ -s_{\theta } & s_{\psi }c_{\theta } & c_{\phi }c_{\theta } \end{array}\right]$$

where c is cos and s is sin.

The transition of the body frame from the angular velocity $\omega ={\left(p,\;q,\;r\right)}^{T}$ to the derivative of the attitude angles $\dot{\zeta }={\left(\dot{\phi },\;\dot{\theta },\;\dot{\psi }\right)}^{T}$ is

(2) $$\left[\begin{array}{c} p\\ q\\ r \end{array}\right]=\left[\begin{array}{ccc} 1 & 0 & s_{\theta }\\ 0 & c_{\phi } & s_{\phi }c_{\theta }\\ 0 & -s_{\phi } & c_{\phi }c_{\theta } \end{array}\right]\left[\begin{array}{c} \dot{\phi }\\ \dot{\theta }\\ \dot{\psi } \end{array}\right]$$

where p, q, and *r* are the angular velocity components of the UAV expressed in the fixed-body frame, representing rotation rates around the *x*-(roll), *y*-(pitch), and *z*-(yaw) axes, respectively. $\dot{\phi },\;\dot{\theta },$ and $\dot{\psi }$ denote the time derivatives of the Euler angles ϕ (roll), θ (pitch), and ψ (yaw), which describe the UAV’s orientation in the inertial (world) frame.

### 2.2. Dynamic Model (Rotational Motion)

The dynamic equations are derived based on the Newton–Euler method [14].

(3) $$J_{b}\dot{\omega }+\omega \times \left(J_{b}\omega \right)=M_{b}-M_{a}$$

where $J_{b}\left[3\times 3\right]$ is the diagonal moment of the inertia matrix, with $J_{xx},\;J_{yy}$, and $J_{zz}$ representing the moment of inertia around the x-axis, y-axis, and z-axis, respectively; $M_{b}$ is the moments of the body frame; $M_{a}$ is the drag moment; and ω and $\dot{\omega }$ are the angular velocity and angular acceleration, respectively.

The aerodynamic force and moment are shown as in [15].

(4) $$F_{i}=\frac{1}{2}\rho AC_{f}\omega_{i}^{2}=k_{f}\omega_{i}^{2}\;\mathrm{and}\;M_{i}=\frac{1}{2}\rho AC_{D}\omega_{i}^{2}=k_{m}\omega_{i}^{2}$$

where ρ is the density of air, A is the area of the blade, $C_{f}$ and $C_{D}$ are aerodynamic coefficients, *i* is the index of the rotor number, $\omega_{i}$ is the angular velocity of rotor i, and $k_{f}$ and $k_{m}$ are the aerodynamic constants for force and moment, respectively, and can be determined experimentally.

(5) $$M_{a}=\left[\begin{array}{ccc} k_{m} & 0 & 0\\ 0 & k_{m} & 0\\ 0 & 0 & k_{m} \end{array}\right]\left[\begin{array}{c} \dot{\phi }\\ \dot{\theta }\\ \dot{\psi } \end{array}\right]$$

As seen in Figure 1, the moments ($M_{x},\;M_{y},$ and $M_{z}$) around the x-axis, y-axis, and z-axis can be determined as the following matrix form:

(6) $$M_{b}=\left[\begin{array}{c} M_{x}\\ M_{y}\\ M_{z} \end{array}\right]=\left[\begin{array}{c} lk_{f}\left(\omega_{4}^{2}-\omega_{2}^{2}\right)\\ lk_{f}\left(\omega_{1}^{2}-\omega_{3}^{2}\right)\\ k_{m}\left(\omega_{1}^{2}-\omega_{2}^{2}+\omega_{3}^{2}-\omega_{4}^{2}\right) \end{array}\right]$$

where *l* is the length of the quadrotor arm.

We can substitute Equations (5) and (6) into Equation (3) to get the following:

(7) $$\begin{array}{cc} \; & \left[\begin{array}{ccc} J_{xx} & 0 & 0\\ 0 & J_{yy} & 0\\ 0 & 0 & J_{zz} \end{array}\right]\left[\begin{array}{c} \ddot{\phi }\\ \ddot{\theta }\\ \ddot{\psi } \end{array}\right]+\left[\begin{array}{c} \dot{\phi }\\ \dot{\theta }\\ \dot{\psi } \end{array}\right]\times \left(\left[\begin{array}{ccc} J_{xx} & 0 & 0\\ 0 & J_{yy} & 0\\ 0 & 0 & J_{zz} \end{array}\right]\left[\begin{array}{c} \dot{\phi }\\ \dot{\theta }\\ \dot{\psi } \end{array}\right]\right)\\ \; & =\left[\begin{array}{c} lk_{f}\left(\omega_{4}^{2}-\omega_{2}^{2}\right)\\ lk_{f}\left(\omega_{1}^{2}-\omega_{3}^{2}\right)\\ k_{m}\left(\omega_{1}^{2}-\omega_{2}^{2}+\omega_{3}^{2}-\omega_{4}^{2}\right) \end{array}\right]-\left[\begin{array}{ccc} k_{m} & 0 & 0\\ 0 & k_{m} & 0\\ 0 & 0 & k_{m} \end{array}\right]\left[\begin{array}{c} \dot{\phi }\\ \dot{\theta }\\ \dot{\psi } \end{array}\right] \end{array}$$

We can simplify Equation (7) to get the following:

(8) $$\left[\begin{array}{c} J_{xx}\ddot{\phi }\\ J_{yy}\ddot{\theta }\\ J_{zz}\ddot{\psi } \end{array}\right]+\left[\begin{array}{c} \dot{\theta }J_{zz}\dot{\psi }-\dot{\psi }J_{yy}\dot{\theta }\\ \dot{\psi }J_{xx}\dot{\phi }-\dot{\phi }J_{zz}\dot{\psi }\\ \dot{\phi }J_{yy}\dot{\theta }-\dot{\theta }J_{xx}\dot{\phi } \end{array}\right]=\left[\begin{array}{c} lk_{f}\left(\omega_{4}^{2}-\omega_{2}^{2}\right)\\ lk_{f}\left(\omega_{1}^{2}-\omega_{3}^{2}\right)\\ k_{m}\left(\omega_{1}^{2}-\omega_{2}^{2}+\omega_{3}^{2}-\omega_{4}^{2}\right) \end{array}\right]-\left[\begin{array}{ccc} k_{m} & 0 & 0\\ 0 & k_{m} & 0\\ 0 & 0 & k_{m} \end{array}\right]\left[\begin{array}{c} \dot{\phi }\\ \dot{\theta }\\ \dot{\psi } \end{array}\right]$$

### 2.3. Dynamic Model (Translation)

The body frame’s translational motion was derived based on Newton’s law [16].

(9) $$m\;\ddot{\xi }=F_{g}+R\;F_{b}$$

where *m* is the mass of the quadrotor UAV, $\xi ={\left[x,\;y,\;z\right]}^{T}$ is the position vector of the quadrotor, $F_{b}$ is the non-gravitational disturbing forces acting on the quadrotor in the b-frame, and $F_{g}$ represents the gravitational force acting on the quadrotor.

(10) $$F_{g}=\left[\begin{array}{c} 0\\ 0\\ -mg \end{array}\right]$$

The thrust forces $F_{b}$ can be written in matrix form as

(11) $$F_{b}=\left[\begin{array}{c} 0\\ 0\\ k_{f}\left(\omega_{1}^{2}+\omega_{2}^{2}+\omega_{3}^{2}+\omega_{4}^{2}\right) \end{array}\right]$$

So, we can substitute Equations (10) and (11) into Equation (9) to get the following:

(12) $$\begin{array}{cc} m\left[\begin{array}{c} \ddot{x}\\ \ddot{y}\\ \ddot{z} \end{array}\right]\; & =\left[\begin{array}{c} 0\\ 0\\ -mg \end{array}\right]+\\ \; & \;\left[\begin{array}{ccc} c_{\theta }c_{\phi } & -c_{\psi }s_{\phi }+s_{\theta }c_{\phi }s_{\psi } & s_{\phi }s_{\psi }+c_{\phi }s_{\theta }c_{\psi }\\ c_{\theta }s_{\phi } & c_{\psi }c_{\phi }+s_{\theta }s_{\phi }s_{\psi } & -c_{\psi }s_{\phi }+c_{\phi }s_{\theta }s_{\psi }\\ -s_{\theta } & s_{\psi }c_{\theta } & c_{\phi }c_{\theta } \end{array}\right]\left[\begin{array}{c} 0\\ 0\\ k_{f}\left(\omega_{1}^{2}+\omega_{2}^{2}+\omega_{3}^{2}+\omega_{4}^{2}\right) \end{array}\right] \end{array}$$

The quadrotor has four rotors that are used to control the machine by acting on their rotational speeds, which are represented by ($U_{1}$, $U_{2},\;U_{3},$ and $U_{4}$). These control vectors can be expressed as follows [17]:

(13) $$u=\left[\begin{array}{cccc} U_{1} & U_{2} & U_{3} & U_{4} \end{array}\right]$$

(14) $$U_{1}=k_{f}\left(\omega_{1}^{2}+\omega_{2}^{2}+\omega_{3}^{2}+\omega_{4}^{2}\right)$$

(15) $$U_{2}=k_{f}\left(-\omega_{2}^{2}+\omega_{4}^{2}\right)$$

(16) $$U_{3}=k_{f}\left(\omega_{1}^{2}-\omega_{3}^{2}\right)$$

(17) $$U_{4}=k_{m}\left(\omega_{1}^{2}-\omega_{2}^{2}+\omega_{3}^{2}-\omega_{4}^{2}\right)$$

and the following applies:

$U_{1}$ controls the quadrotor’s height and rate of change (z, $\dot{z}$);
$U_{2}$ controls the roll angle to rotate and change at a constant rate ($\phi ,\dot{\phi }$);
$U_{3}$ controls the rotation and rate change of the pitch angle ($\theta ,\dot{\theta }$).
$U_{4}$ controls the yaw motion and rate change ($\psi ,\;\dot{\psi }$).

The simplified model of the quadrotor UAV can be obtained as follows:

(18) $$\ddot{x}=\frac{U_{1}}{m}\left(c_{\phi }s_{\theta }c_{\psi }+s_{\phi }s_{\psi }\right)$$

(19) $$\ddot{y}=\frac{U_{1}}{m}\left(c_{\phi }s_{\theta }s_{\psi }-s_{\phi }c_{\psi }\right)$$

(20) $$\ddot{z}=\frac{U_{1}}{m}\left(c_{\phi }c_{\theta }\right)-g$$

(21) $$\ddot{\phi }=\frac{1}{J_{xx}}\left(\dot{\theta }\dot{\psi }\left(J_{yy}-J_{zz}\right)+lU_{2}\right)$$

(22) $$\ddot{\theta }=\frac{1}{J_{yy}}\left(\dot{\phi }\dot{\psi }\left(J_{zz}-J_{xx}\right)+lU_{3}\right)$$

(23) $$\ddot{\psi }=\frac{1}{J_{zz}}\left(\dot{\phi }\dot{\theta }\left(J_{xx}-J_{yy}\right)+U_{4}\right)$$

Finally, the mathematical model of the quadrotor can be represented in the state space method as follows:

$$\dot{x}=f\left(x,u\right)$$

where the state vector $x={\left[x_{1}\;x_{2}\;\ldots \;x_{12}\right]}^{T}={\left[x\;\dot{x}\;y\;\dot{y}\;z\;\dot{z}\;\phi \;\dot{\phi }\;\theta \;\dot{\theta }\;\psi \;\dot{\psi }\right]}^{T},$ and $u={\left[U_{1}\;U_{2}\;U_{3}\;U_{4}\right]}^{T}$.

(24) $$\begin{array}{cc} {\dot{x}}_{1} & =x_{2},\\ {\dot{x}}_{2} & =\frac{U_{1}}{m}\left(c_{x_{7}}\;s_{x_{9}}\;c_{x_{11}}+s_{x_{7}}\;s_{x_{11}}\right),\\ {\dot{x}}_{3} & =x_{4},\\ {\dot{x}}_{4} & =\frac{U_{1}}{m}\left(c_{x_{7}}\;s_{x_{9}}\;s_{x_{11}}-s_{x_{7}}\;c_{x_{11}}\right),\\ {\dot{x}}_{5} & =x_{6},\\ {\dot{x}}_{6} & =\frac{U_{1}}{m}\left(c_{x_{7}}\;c_{x_{9}}\right)-g,\\ {\dot{x}}_{7} & =x_{8},\\ {\dot{x}}_{8} & =q_{1}\;x_{10}x_{12}+q_{2}\;U_{2},\\ {\dot{x}}_{9} & =x_{10},\\ {\dot{x}}_{10} & =q_{3}\;x_{8}x_{12}+q_{4}\;U_{3},\\ {\dot{x}}_{11} & =x_{12},\\ {\dot{x}}_{12} & =q_{5}\;x_{8}x_{10}+q_{6}\;U_{4}. \end{array}$$

where

(25) $$\begin{array}{cccc} q_{1} & =\frac{J_{yy}-J_{zz}}{J_{xx}}, & \;q_{2} & =\frac{l}{J_{xx}},\\ q_{3} & =\frac{J_{zz}-J_{xx}}{J_{yy}}, & \;q_{4} & =\frac{l}{J_{yy}},\\ q_{5} & =\frac{J_{xx}-J_{yy}}{J_{zz}}, & \;q_{6} & =\frac{1}{J_{zz}}. \end{array}$$

## 3. Fractional-Order PID Controller

**Figure 2:**
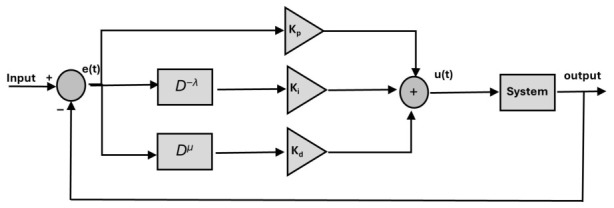
Block diagram of FOPID control [17].

In general, a PID controller based on a fractional order is known as a Fractional-Order PID (FOPID). In addition to the three gains of the traditional PID controller, FOPID has two more gains applied to integrals and derivatives terms. As a result, it offers greater freedom in controller design and system modeling, as well as enables a wide range of applications. The general equation for the FOPID controller is shown in Figure 2 [17,18]:

(26) $$u\left(t\right)=k_{p}e\left(t\right)+k_{i}D^{-\lambda }e\left(t\right)+k_{d}D^{\mu }e\left(t\right)$$

where the proportional, integral, and derivative gains are $k_{p},\;k_{i}$ and $k_{d}$; the orders of the integral and derivative terms are indicated by λ and μ; and the signal errors are represented by *e(t)*.

### 3.1. Design of the FOPID Controller for the Quadrotor Altitude System

The FOPID was employed to generate the control signal $U_{1}$, which is used to control the altitude of the quadrotor system.

(27) $$U_{1}\left(t\right)=k_{pz}e_{z}\left(t\right)+k_{iz}D^{-\lambda }e_{z}\left(t\right)+k_{dz}D^{\mu }e_{z}\left(t\right)$$

### 3.2. Design of the FOPID Controller for the Quadrotor Attitude System

The attitude (roll, pitch, and yaw angles) of the UAV is controlled by three signals.

The control signal $U_{2}$ is produced by the FOPID controller for the roll angle (ϕ) associated with error $e_{\phi }\left(t\right)$.

(28) $$U_{2}\left(t\right)=k_{p\phi }e_{\phi }\left(t\right)+k_{i\phi }D^{-\lambda }e_{\phi }\left(t\right)+k_{d\phi }D^{\mu }e_{\phi }\left(t\right)$$

The next control signal $U_{3}$ is generated to control the pitch angle (θ) associated with error $e_{\theta }\left(t\right)$.

(29) $$U_{3}\left(t\right)=k_{p\theta }e_{\phi }\left(t\right)+k_{i\theta }D^{-\lambda }e_{\theta }\left(t\right)+k_{d\theta }D^{\mu }e_{\theta }\left(t\right)$$

$U_{4}$ (t) signal is used to control the yaw angle (ψ) associated with error $e_{\psi }\left(t\right)$; the equation for the controller is

(30) $$U_{4}\left(t\right)=k_{p\psi }e_{\psi }\left(t\right)+k_{i\psi }D^{-\lambda }e_{\psi }\left(t\right)+k_{d\psi }D^{\mu }e_{\psi }\left(t\right)$$

where the subscripts (ϕ, θ, and ψ) indicates the gains of the rotating angles, while the superscripts (λ and μ) indicate the integral and the derivative orders, respectively.

## 4. Optimal FOPID Control Using PSO

James Kennedy and Russell C. Eberhart [19] presented PSO, an innovative computational intelligence method inspired by interaction patterns observed in nature, especially in bird flocking. A possible solution, referred to as a particle, moves through the search space while exchanging data with other elements in PSO. Two essential variables define each particle:

$x_{id}$ is the particle’s current position in the search space.
$v_{id}$ is the particle’s current velocity.

The position where particle *i* has achieved the lowest objective function value (in a minimization problem) is known as the personal best value $p_{id}$. The global best, represented by $p_{gd}$, is the best position discovered by the entire swarm. Particles use predetermined equations to update their positions and velocities at every iteration. Equation (31) is used to update the velocity, while Equation (32) is used to update the position. The positive acceleration coefficients $c_{1}$ and $c_{2}$ in these equations are multiplied by random values in the interval [0,1]. To balance both exploration and extraction, the inertia weight *w* decreases linearly from 0.9 to 0.4 throughout the algorithm. Particle velocities are limited to predetermined minimum and maximum values.

(31) $$v_{id}=w\;v_{id}+c_{1}\;\mathrm{rand}\left(1\right)\;\left(p_{id}-x_{id}\right)+c_{2}\;\mathrm{rand}\left(2\right)\;\left(p_{gd}-x_{id}\right)$$

(32) $$x_{i+1}=x_{i}+v_{id}$$

The PSO technique identifies the population best and the global best for any (*i*) particle [20]. It is important to note that Equations (31) and (32) are used to modify each particle’s velocity and position within the swarm.

The steps of the PSO algorithm are as follows:

1. Initialize each particle in the swarm with an arbitrary position.
2. Evaluate the fitness of each particle using the chosen fitness function (here, the mean square error).
3. Compare each particle’s fitness with its personal best to identify the superior current value and update the personal best accordingly.
4. Determine the global best as the particle with the best fitness value and record its position as $p_{gd}$.
5. Update the velocity and position of each particle using Equations (31) and (32).
6. Repeat steps 2–5 until either the maximum number of iterations is reached or the fitness value meets a satisfactory accuracy result.

## 5. Lyapunov-Based Backstepping Design

Inaccurate modeling, parametric uncertainties, and external disturbances are the reasons for the changes in the dynamic model. In this section, a Lyapunov-based backstepping method (LYP) was used to control a quadrotor UAV, ensuring robust and accurate control. This method updates the control parameter settings of the UAV dynamic model in real time [21].

Initially, a positive-definite Lyapunov function with a negative semi-definite time derivative is defined for the tracking error as follows [22,23]:

The desired trajectory is $z_{d}$, and the trajectory error is defined as

(33) $$\begin{array}{cc} z_{d} & =x_{5d} \end{array}$$

(34) $$\begin{array}{cc} e_{5} & =z_{d}-z \end{array}$$

(35) $$\begin{array}{cc} {\dot{e}}_{5} & ={\dot{z}}_{d}-\dot{z} \end{array}$$

A positive definite Lyapunov function $V_{1}$ is defined as

(36) $$V_{1}=\frac{1}{2}e_{5}^{2}$$

Then, the derivative of $V_{1}$ is

(37) $${\dot{V}}_{1}=e_{5}{\dot{e}}_{5}=e_{5}\left({\dot{z}}_{d}-\dot{z}\right)$$

The derivative $\dot{z}$ is used as an intermediate command to satisfy the Lyapunov function:

(38) $${\dot{V}}_{1}=-k_{1}e_{5}^{2}$$

with $k_{1}>0$.

For this purpose, we define a virtual command $z_{2d}$:

(39) $$\begin{array}{cc} z_{2d} & ={\dot{z}}_{d}+k_{1}e_{5} \end{array}$$

(40) $$\begin{array}{cc} {\dot{z}}_{2d} & ={\ddot{z}}_{d}+k_{1}{\dot{e}}_{5} \end{array}$$

At this stage, a new error is generated:

(41) $$\begin{array}{cc} e_{6} & =z_{2d}-\dot{z}={\dot{z}}_{d}+k_{1}e_{5}-\dot{z} \end{array}$$

(42) $$\begin{array}{cc} {\dot{e}}_{6} & ={\ddot{z}}_{d}+k_{1}{\dot{e}}_{5}-\ddot{z} \end{array}$$

Using Equations (35) and (41), we find that

(43) $$\begin{array}{cc} e_{6} & ={\dot{e}}_{5}+k_{1}e_{5} \end{array}$$

(44) $$\begin{array}{cc} {\dot{e}}_{5} & =e_{6}-k_{1}e_{5} \end{array}$$

To eliminate this error, the Lyapunov candidate function $V_{1}$ is augmented by another term:

(45) $$V_{2}=\frac{1}{2}\left(e_{5}^{2}+e_{6}^{2}\right)$$

Then, the derivative of $V_{2}$ is

(46) $${\dot{V}}_{2}=e_{5}{\dot{e}}_{5}+e_{6}{\dot{e}}_{6}$$

Using Equations (42) and (44) in Equation (46), we get

(47) $$\begin{array}{cc} {\dot{V}}_{2} & =e_{5}\left(e_{6}-k_{1}e_{5}\right)+e_{6}\left({\ddot{z}}_{d}+k_{1}{\dot{e}}_{5}-\ddot{z}\right) \end{array}$$

(48) $$\begin{array}{cc} \; & =-k_{1}e_{5}^{2}+e_{6}\left({\ddot{z}}_{d}-\ddot{z}+k_{1}\left(e_{6}-k_{1}e_{5}\right)+e_{5}\right) \end{array}$$

Using Equation (33) and replacing $\ddot{z}$ in Equation (48), we obtain

(49) $${\dot{V}}_{2}=-k_{1}e_{5}^{2}+e_{6}\left({\ddot{z}}_{d}-\left(U_{1}/\left(mc_{x_{7}}c_{x_{9}}\right)-g\right)+k_{1}\left(e_{6}-k_{1}e_{5}\right)+e_{5}\right)$$

In order to satisfy the Lyapunov condition ${\dot{V}}_{2}\leq 0$, the following expression is used:

(50) $${\ddot{z}}_{d}-\left(\frac{U_{1}}{mc_{x_{7}}c_{x_{9}}}-g\right)+k_{1}\left(e_{6}-k_{1}e_{5}\right)+e_{5}=-k_{2}e_{6}$$

with $k_{2}>0$.

By replacing the error dynamics and the selected virtual and real control laws into the time derivative of the Lyapunov function (Equation (49)) and by suitably selecting the control input (Equation (50)) to eliminate nonlinear and cross-coupled effects, the derivative is reduced to

$${\dot{V}}_{2}=-k_{1}e_{5}^{2}+-k_{2}e_{6}^{2}$$

This ensures asymptotic stability of the system or ${\dot{V}}_{2}\leq 0$.

(51) $$U_{1}=\frac{m}{c_{x_{7}}c_{x_{9}}}\left[{\ddot{z}}_{d}-g-k_{1}\left(e_{6}-k_{1}e_{5}\right)-e_{5}-k_{2}e_{6}\right]$$

Following the same procedure, we obtain

(52) $$\begin{array}{cc} U_{2} & =\frac{1}{q_{2}}\left[{\ddot{\phi }}_{d}-q_{1}x_{10}x_{12}+k_{3}\left(e_{8}-k_{3}e_{7}\right)+e_{7}+k_{4}e_{8}\right] \end{array}$$

(53) $$\begin{array}{cc} U_{3} & =\frac{1}{q_{4}}\left[{\ddot{\theta }}_{d}-q_{3}x_{8}x_{12}+k_{5}\left(e_{10}-k_{5}e_{9}\right)+e_{9}+k_{6}e_{10}\right] \end{array}$$

(54) $$\begin{array}{cc} U_{4} & =\frac{1}{q_{6}}\left[{\ddot{\psi }}_{d}-q_{5}x_{8}x_{10}+k_{7}\left(e_{12}-k_{7}e_{11}\right)+e_{11}+k_{8}e_{12}\right] \end{array}$$

## 6. Optimal Gains of Lyapunov-Based Control

A controller based on Equations (51)–(54) was intended to stabilize the altitude and attitude of the UAV. The coefficients $k_{i}$, where *i* = 1 to 8, are gains of the controller in Equations (51)–(54). The coefficients should be positive to meet the stability criteria of the Lyapunov equations. These parameters are tuned using the PSO technique to select the optimal parameter values. This method is used to find gains that provide the best control performance while meeting the Lyapunov stability criteria. PSO with Lyapunov-based control is both helpful and successful for UAV and related applications, as it automates optimal gain selection while ensuring stability [22]. The objective of PSO optimization is to lower the Integral of Absolute Error (IAE) between the desired state and the current state as follows:

(55) $$\mathrm{IAE}=\int_{0}^{t}\left|e\left(t\right)\right|\;dt$$

The LYP algorithm can be summarized as follows:

1. Set up the PSO population.
  - A random swarm of particles is created, where each particle represents a potential set of controller gains.
  - Based on practical and safety factors, each gain is limited to predetermined bounds.
2. The system response is simulated for each particle.
  The tracking error is calculated throughout the simulation time for each particle.
3. Determine the derivative of the Lyapunov function.
  - A Lyapunov candidate function is defined, such as $V=\frac{1}{2}e^{2}.$
  - To guarantee stability, the derivative $\dot{V}$ must satisfy $\dot{V}\leq 0$ at each time step.
4. Evaluate the cost function.
  The IAE is used to calculate the fitness value for each particle using Equation (55).
5. Update the swarm positions.
  - The PSO update rules are used to update each particle’s position and velocity.
  - Steps 2 through 5 are repeated until convergence is achieved or the maximum number of iterations is reached.

## 7. Simulation Results

**Figure 3:**
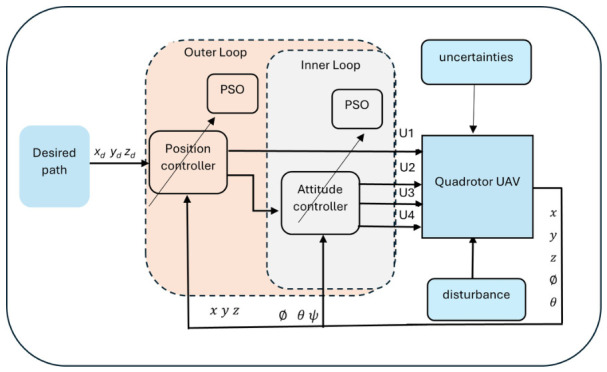
Illustration of the proposed system architecture.

The UAV mathematical model was implemented in MATLAB 2025b. The illustration of the proposed system architecture is shown in Figure 3. This model has two loops: an inner loop for attitude and an outer loop for position. Different controllers were used in this work: PID, FOPID, and a Lyapunov-based backstepping controller. These controllers were compared under the same three conditions: the normal condition, model parameter uncertainties, and external disturbances. The quadrotor parameters that were used in the simulations are shown in Table 1. The following parameters were used for the optimization controller settings in this study: The population/swarm size is 10. The maximum number of iterations is 30, and the range factors for $c_{1}$ and $c_{2}$ are random within [0,1]. The inertia weight factor w is set from 0.9 to 0.4, and the searching ranges for the backstepping parameters are restricted to [0, 20]. Convergence was achieved, and a satisfactory fitness value was obtained using the PSO approach. These findings demonstrate that the PSO method may be fast and efficient at determining the ideal backstepping controller parameters.

The optimal parameters of the position and rotation controllers were calculated, yielding $k_{1}=13.64$, $k_{2}=13.31$, $k_{3}=14.21$, $k_{4}=13.68$, $k_{5}=14.11$, $k_{6}=13.85$, $k_{7}=14.33$, and $k_{8}=13.97$ at a fitness value of 0.131 after 20 iterations.

### 7.1. Performance of UAV Control Under Normal Conditions

**Figure 4:**
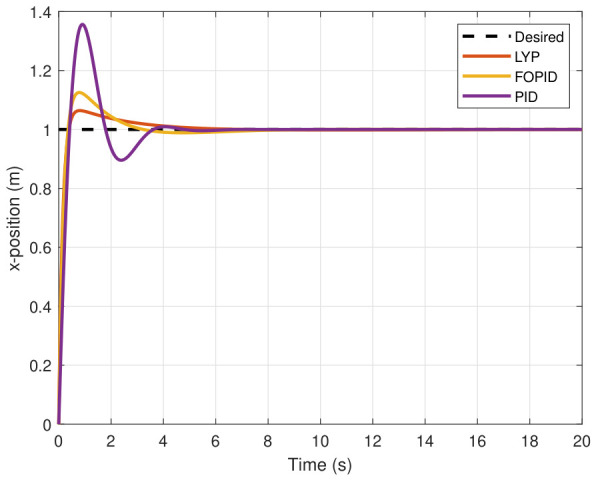
X-position tracking under normal conditions.

**Figure 5:**
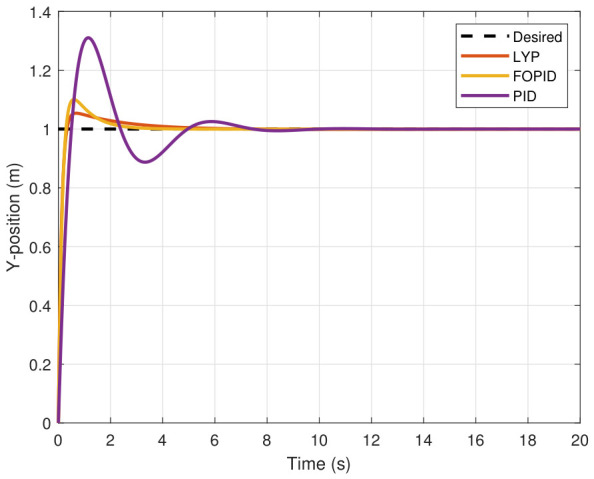
Y-position tracking under normal conditions.

**Figure 6:**
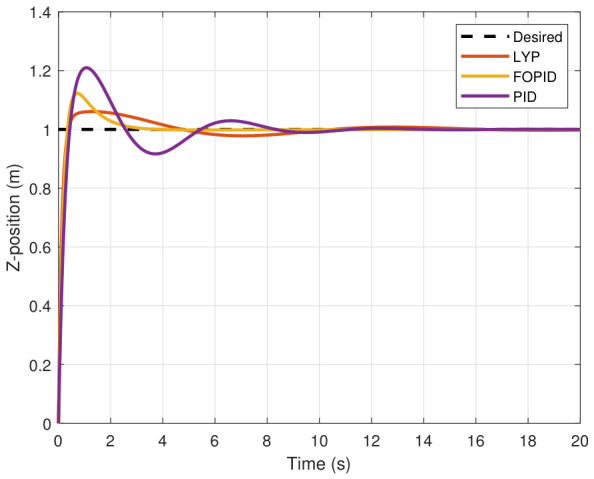
Z-position tracking under normal conditions.

**Figure 7:**
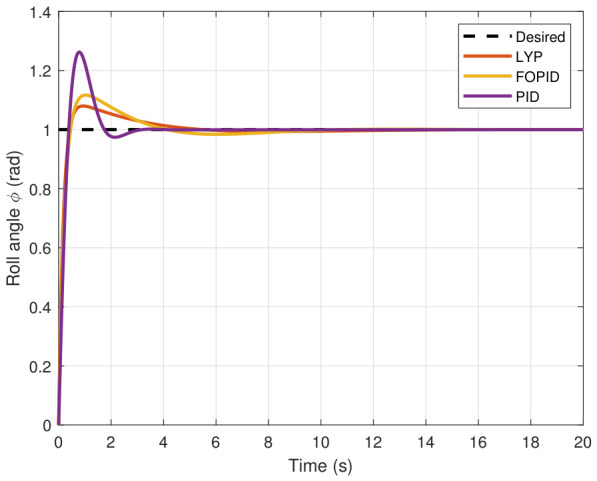
Roll angle tracking under normal conditions.

**Figure 8:**
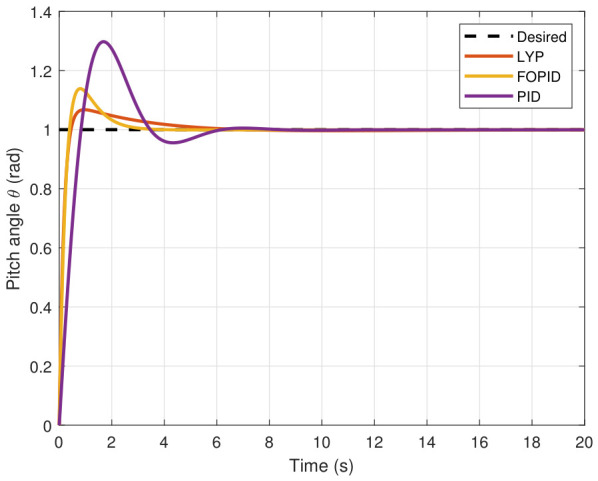
Pitch angle tracking under normal conditions.

**Figure 9:**
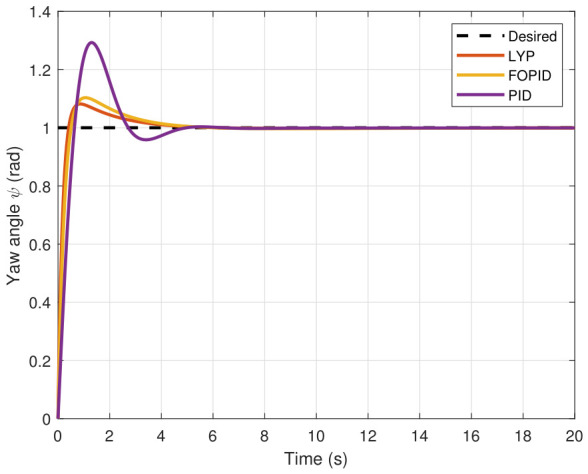
Yaw angle tracking under normal conditions.

The step input was used as the reference for the tracking simulation under nominal conditions (i.e., no disturbances or parameter uncertainties in the UAV). A MATLAB simulation was implemented to track the desired reference signal for each of the x, y, z, ϕ, θ, and ψ states by applying three controllers (PID, FOPID, and Lyapunov-based backstepping). The results are shown in Figure 4, Figure 5, Figure 6, Figure 7, Figure 8 and Figure 9. The trajectories of the FOPID and Lyapunov-based backstepping controllers are observed to converge to the desired trajectories, indicating the controllers’ ability to achieve precise tracking with a transient response. To perform comparative robustness research, the rise time, overshoot, and settling time are collected in Table 2. The overshoot of the Lyapunov-based backstepping controller was lower than that of the FOPID and PID controllers, while the rise time and settling time of the FOPID and Lyapunov-based backstepping controllers were faster, closer, and better than those of the PID controller.

### 7.2. Performance of UAV Control Under Disturbance

**Figure 10:**
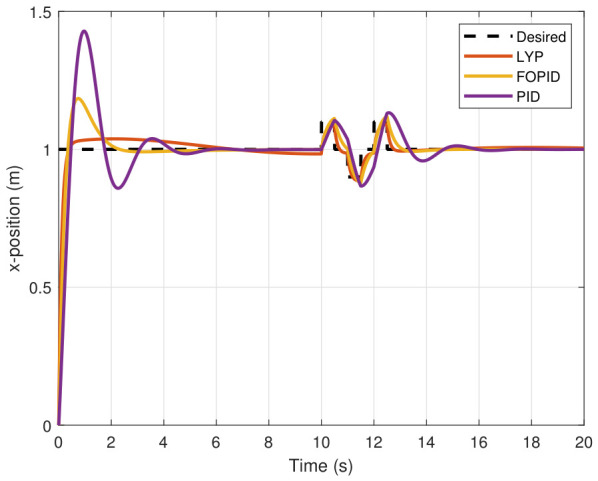
X-position tracking under disturbance.

**Figure 11:**
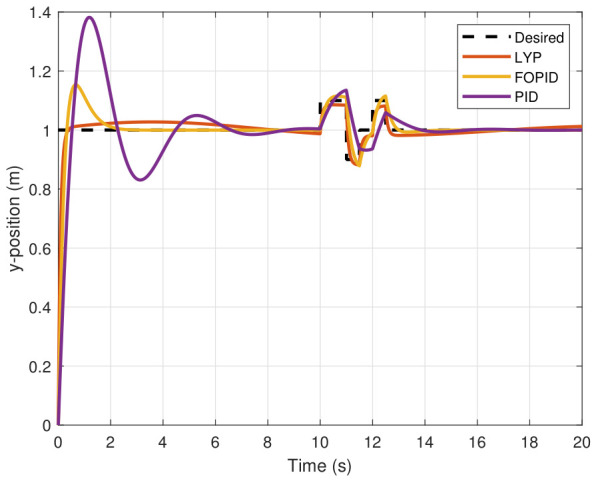
Y-position tracking under disturbance.

**Figure 12:**
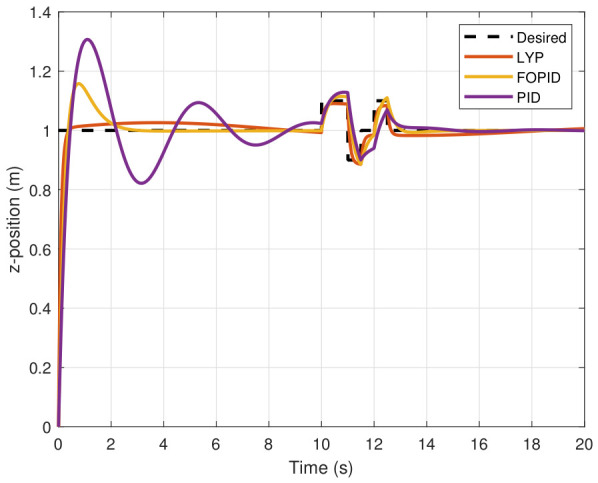
Z-position tracking under disturbance.

**Figure 13:**
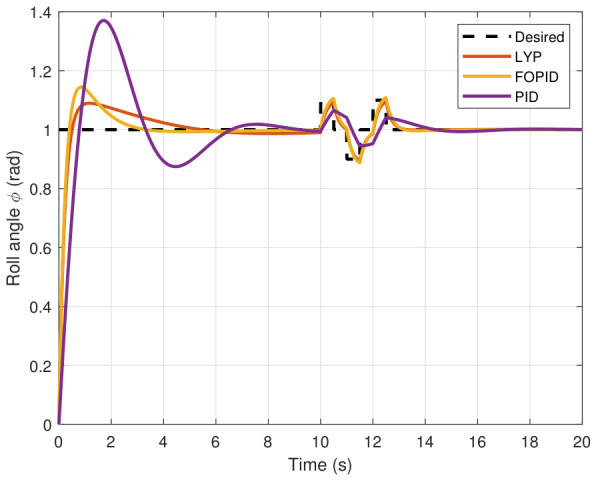
Roll angle tracking under disturbance.

**Figure 14:**
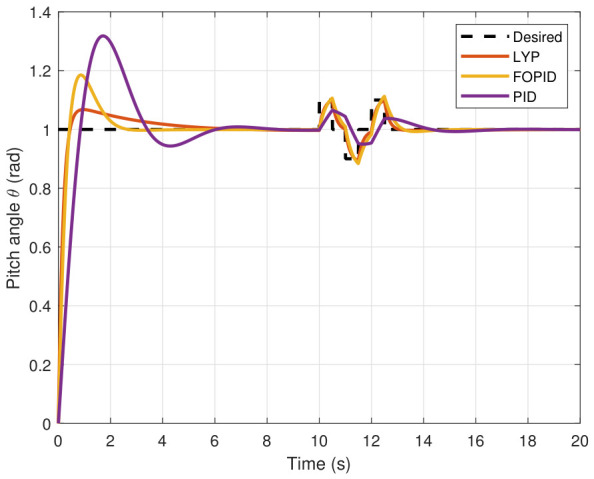
Pitch angle tracking under disturbance.

**Figure 15:**
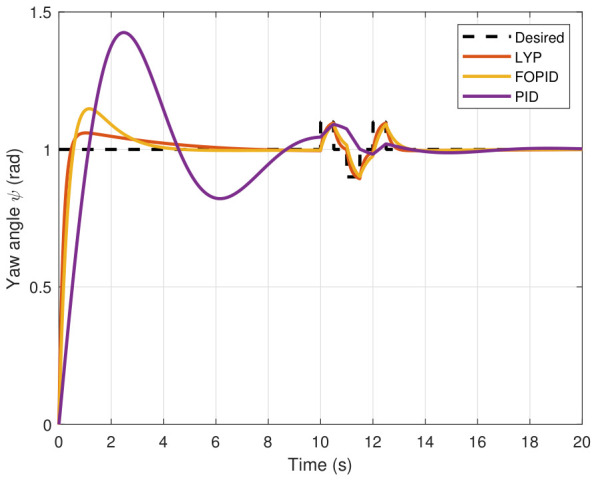
Yaw angle tracking under disturbance.

The simulation results of the FOPID, Lyapunov-based backstepping controllers, and conventional PID control are compared in this study. During testing, the wind velocity is modeled as a square-wave disturbance applied to the system. The results for the position and attitude of the UAV under disturbance are presented in Figure 10, Figure 11, Figure 12, Figure 13, Figure 14 and Figure 15. Each state has effects on the external disturbance starting at 10 s. It appears that both the FOPID and the Lyapunov-based backstepping controllers are less sensitive to external disturbances than PID controllers and can reject disturbances. The UAV’s performance under external disturbance is illustrated in Table 3. The FOPID and Lyapunov-based backstepping controllers achieve comparable and significantly faster rise times, settling times, and overshoots than the PID controller. The rising time remains quick for all controllers, but the settling time is longer during disturbances than during normal conditions. Compared to the normal condition, the overshoot parameter changes slightly under disturbance.

### 7.3. Performance of UAV Control with Parameter Uncertainty

**Figure 16:**
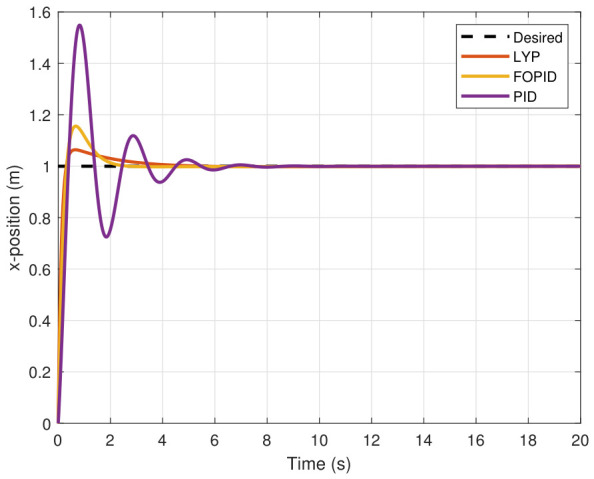
X-position tracking with 50% parameter uncertainty.

**Figure 17:**
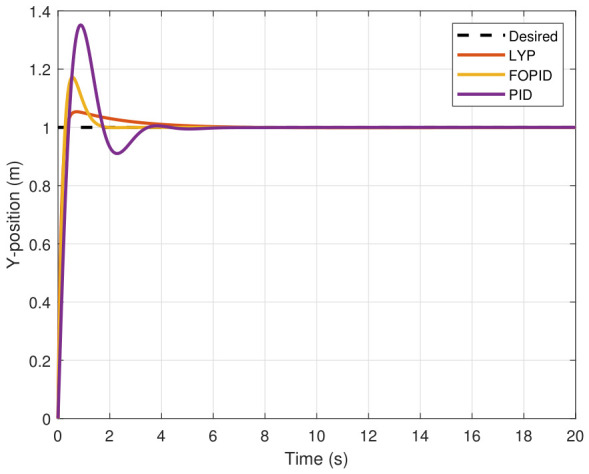
Y-position tracking with 50% parameter uncertainty.

**Figure 18:**
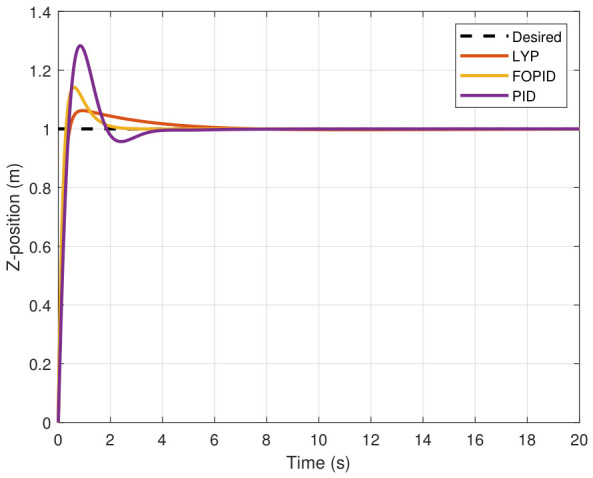
Z-position tracking with 50% parameter uncertainty.

**Figure 19:**
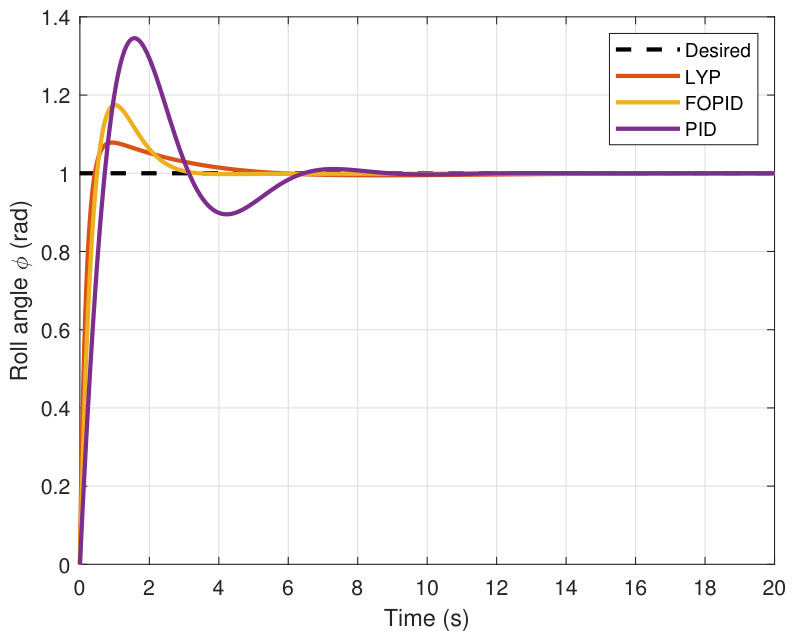
Roll angle tracking with 50% parameter uncertainty.

**Figure 20:**
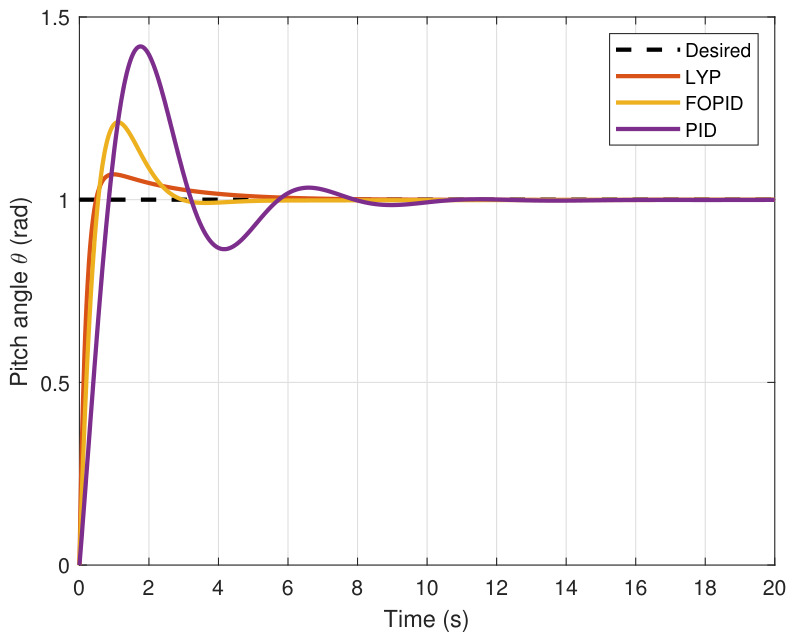
Pitch angle tracking with 50% parameter uncertainty.

**Figure 21:**
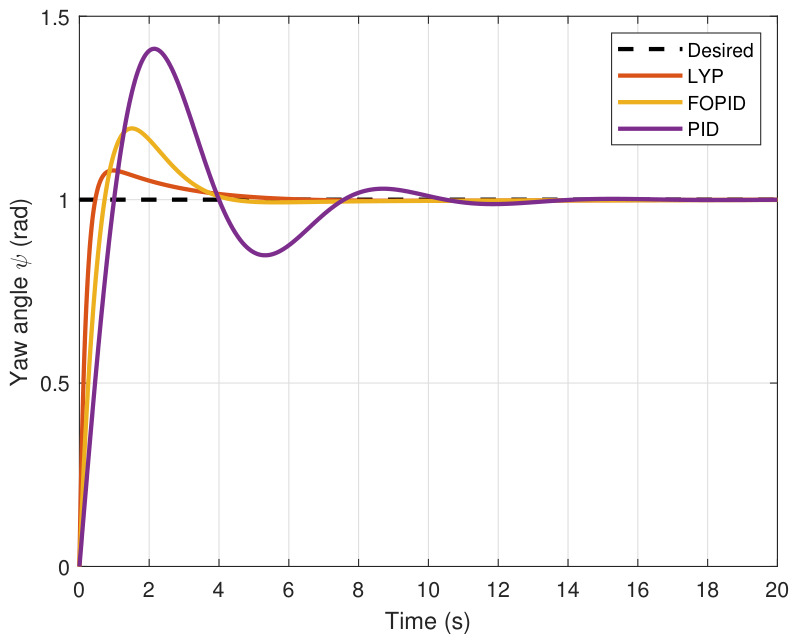
Yaw angle tracking with 50% parameter uncertainty.

This section examines the impact of +50% parameter uncertainty on the quadrotor mass m and moments of inertia $I_{x},\;I_{y}$, and $I_{z}$. The results of simulating the PID, FOPID, and Lyapunov-based backstepping controllers under this type of uncertainty are shown in Figure 16, Figure 17, Figure 18, Figure 19, Figure 20 and Figure 21. These results demonstrate that Lyapunov-based backstepping controllers are more robust than the FOPID and PID controllers. Table 4 shows that Lyapunov-based backstepping controllers achieve quicker settling times. On the other hand, the PID controller exhibits far greater overshoot and longer rise and settling times. The Lyapunov-based backstepping controller exhibits the fastest rise time among all controllers while maintaining overshoot levels comparable to those observed under typical operating conditions, demonstrating excellent resilience to parameter uncertainty.

## 8. Trajectory Tracking Results

The proposed controller is evaluated using two different desired trajectories and compared with other controllers: a circular path and a figure-eight path. The details of the trajectory definitions and initial conditions are provided in the following section.

### 8.1. Tracking the Circle Path

**Figure 22:**
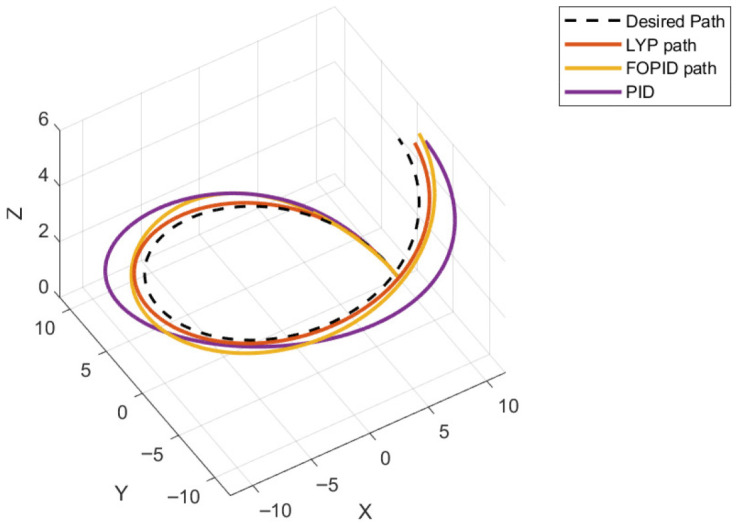
The 3D trajectory of the quadrotor tracking the circular path.

The UAV is commanded to follow a circular trajectory specified by $x_{d}=10\;sin\left(t\right)$ and $y_{d}=10\;cos\left(t\right)$ after starting at rest at point (0, 0). The circular path’s initial condition is chosen to be $x_{0}=\left[10\;m,\;0,\;0,\;0.15\;rad,\;0,\;0\right]$. Figure 22 shows the 3D trajectory tracking result of the quadrotor.

**Figure 23:**
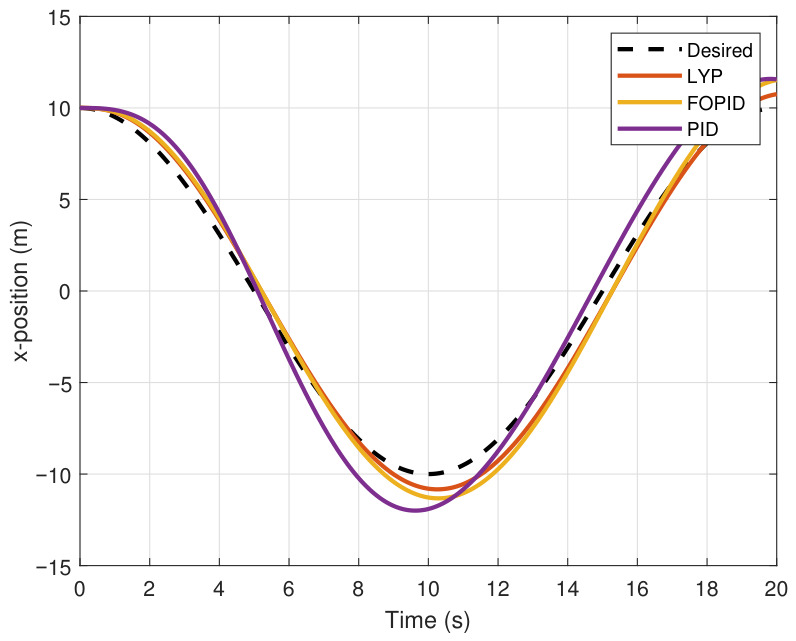
The X-position result of tracking the circular path.

**Figure 24:**
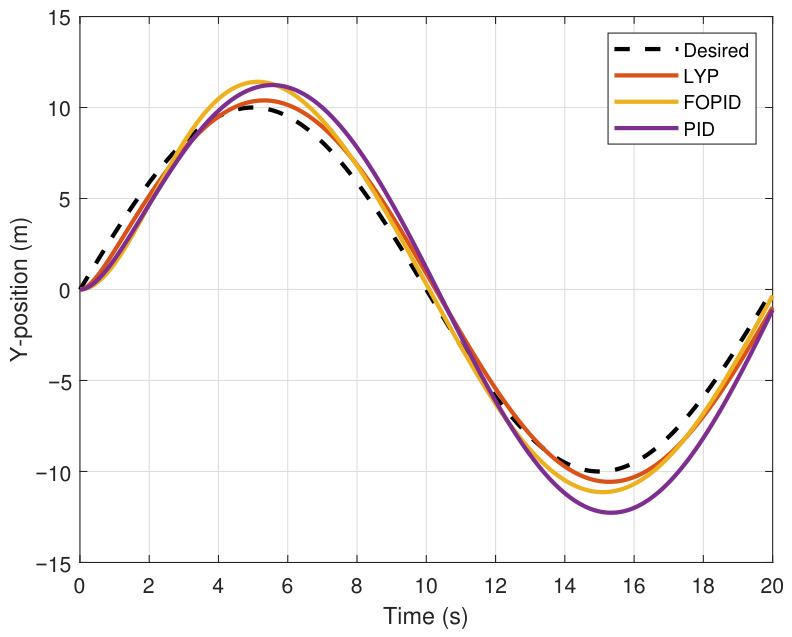
The Y-position result of tracking the circular path.

**Figure 25:**
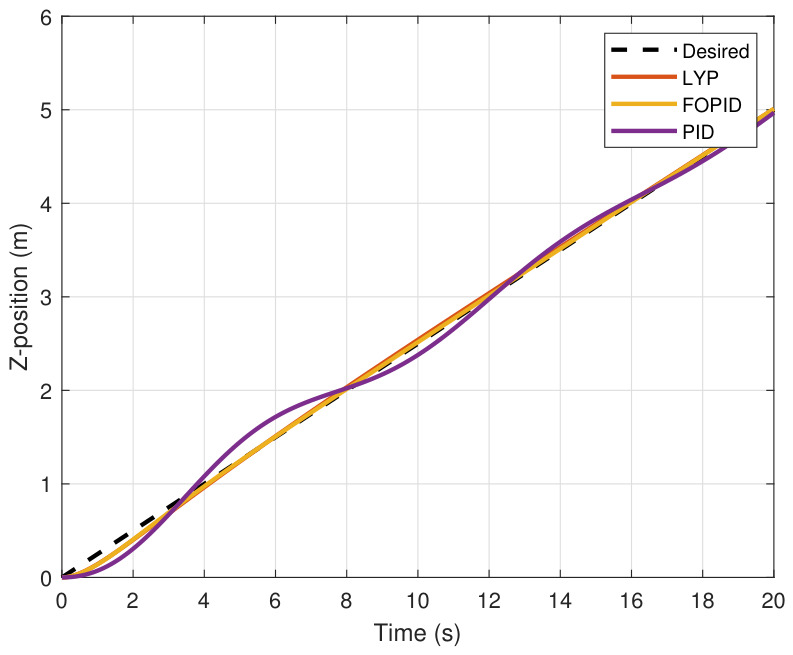
The Z-position result of tracking the circular path.

**Figure 26:**
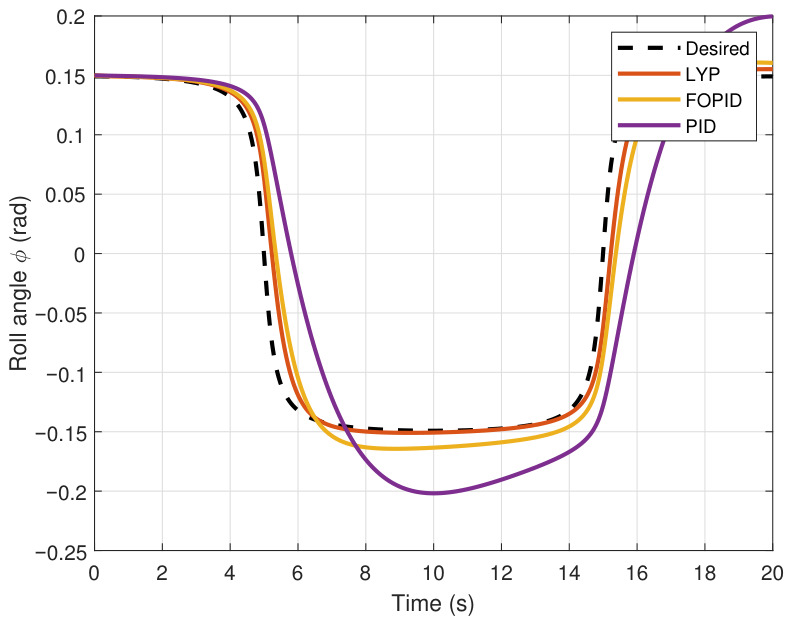
The roll angle result of tracking the circular path.

**Figure 27:**
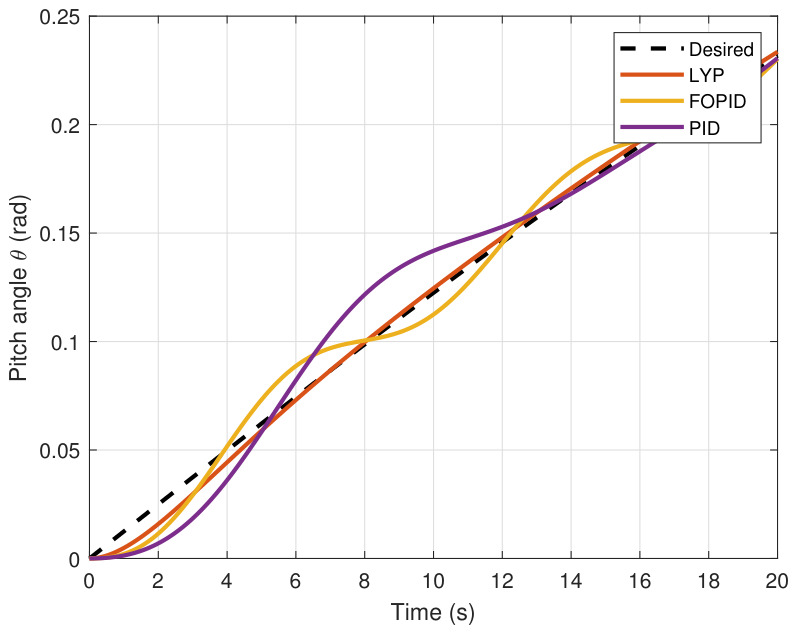
The pitch angle result of tracking the circular path.

**Figure 28:**
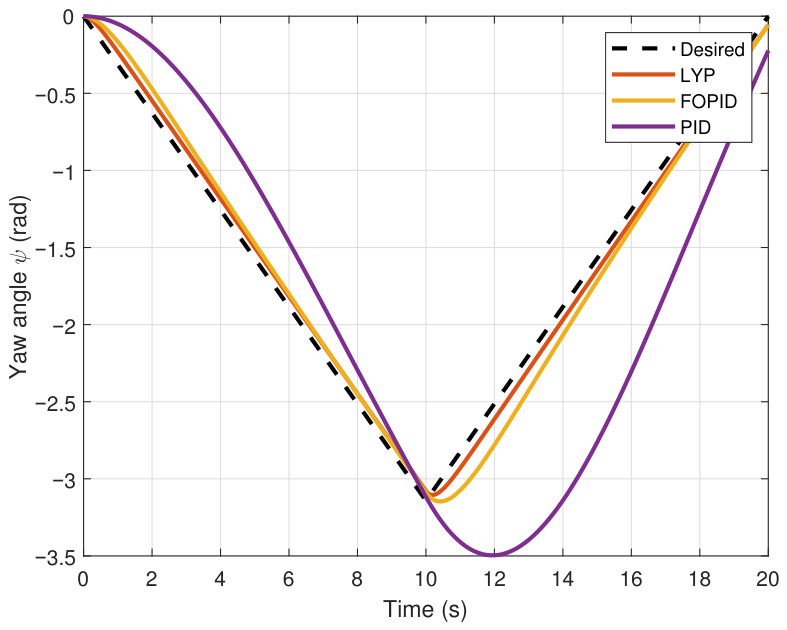
The yaw angle result of tracking the circular path.

**Figure 29:**
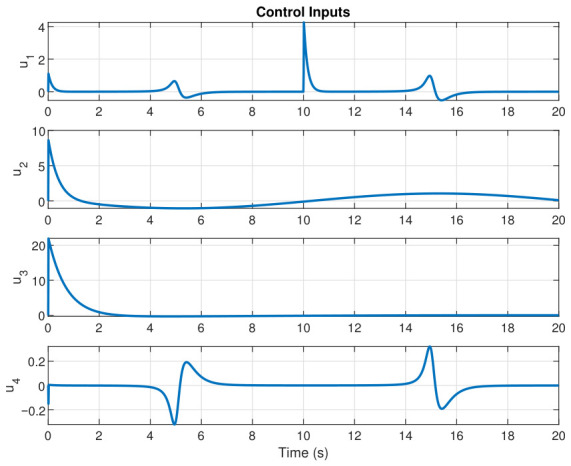
The control input for the circular path.

This section evaluates three controllers: the PID controller, the FOPID controller, and the proposed Lyapunov-based backstepping (LYP) controller. The tracking data are presented in Figure 23, Figure 24, Figure 25, Figure 26, Figure 27, Figure 28 and Figure 29, with LYP in red, FOPID in yellow, PID in purple, and the desired trajectory for position and altitude over a 20-s period represented by a dashed black line. As shown, compared to PID, both LYP and FOPID maintain stable quadrotor attitudes and achieve good tracking accuracy by closely following the desired motion. The four control inputs are shown in Figure 24. After a short transitory time, $U_{2},\;U_{3}$, and $U_{4}$ stay smooth and rapidly stabilize; $U_{1}$ shows the control thrust increment above the hover equilibrium.

The performance indices are summarized using the root mean square error (RMSE) in Table 5. The LYP controller performs better overall, even though the FOPID controller produces acceptable results that are better than those of PID.

### 8.2. Tracking the Figure-Eight Path

**Figure 30:**
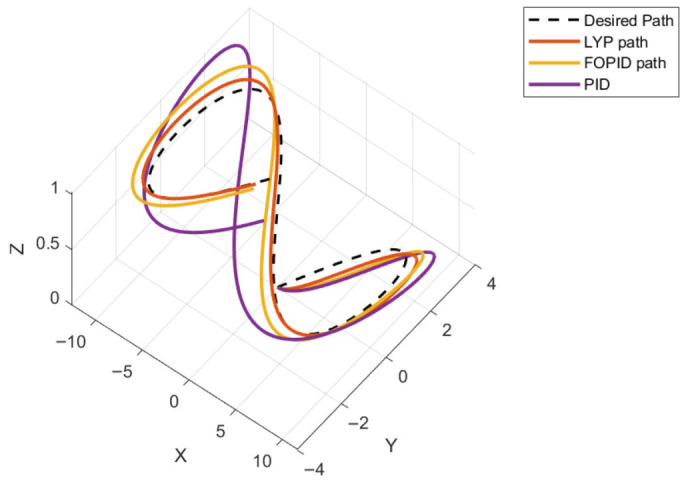
The 3D trajectory of the quadrotor tracking the figure-eight path.

After starting at rest at position (0, 0), the UAV is commanded to follow a circular trajectory defined by the following parameters: $x_{d}=10\;sin\left(t\right),$ $y_{d}=5\;sin\left(t\right)\;cos\left(t\right)$, and $z_{d}=sin\left(t\right)$. Specifically, $x_{0}=[0,\;0,\;0,\;0.15\;$ rad, 0.7 rad, 0.5 rad] is selected as the initial condition for the figure-eight path. Figure 30 shows the three-dimensional (3D) trajectory tracking result for the quadrotor.

**Figure 31:**
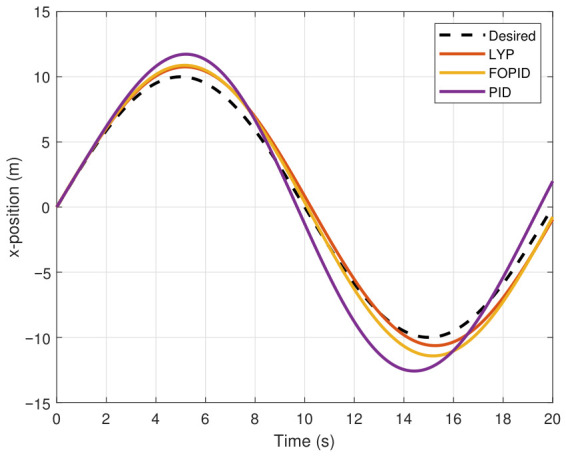
The x-position result of tracking the figure-eight path.

**Figure 32:**
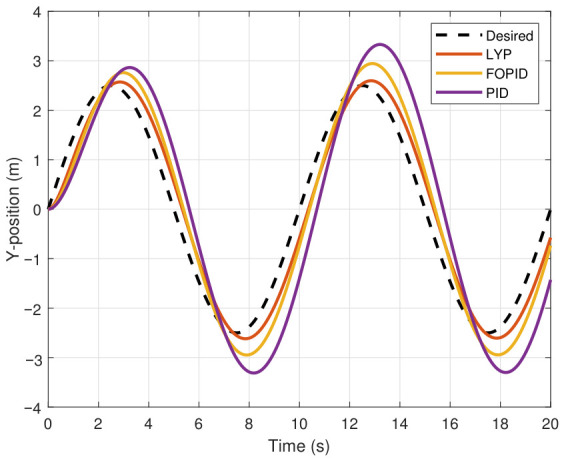
The y-position result of tracking the figure-eight path.

**Figure 33:**
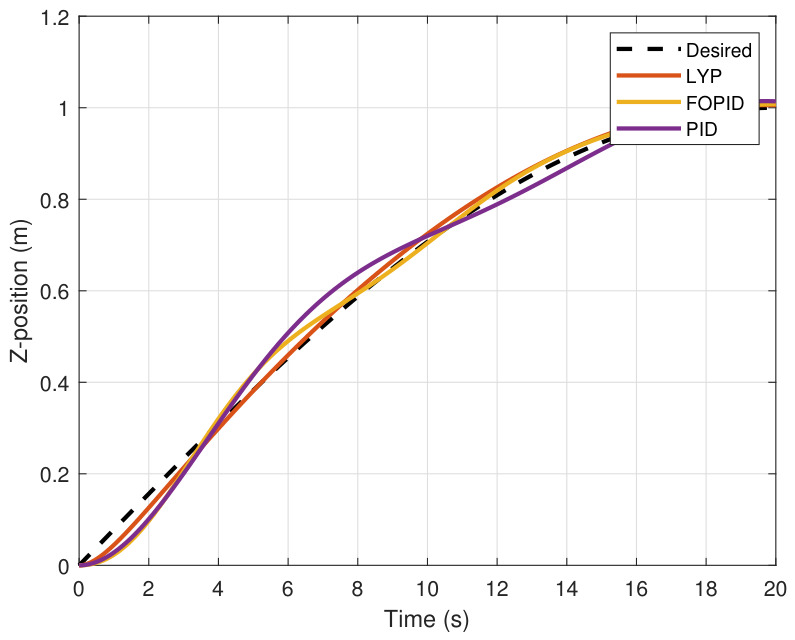
The z-position result of tracking the figure-eight path.

**Figure 34:**
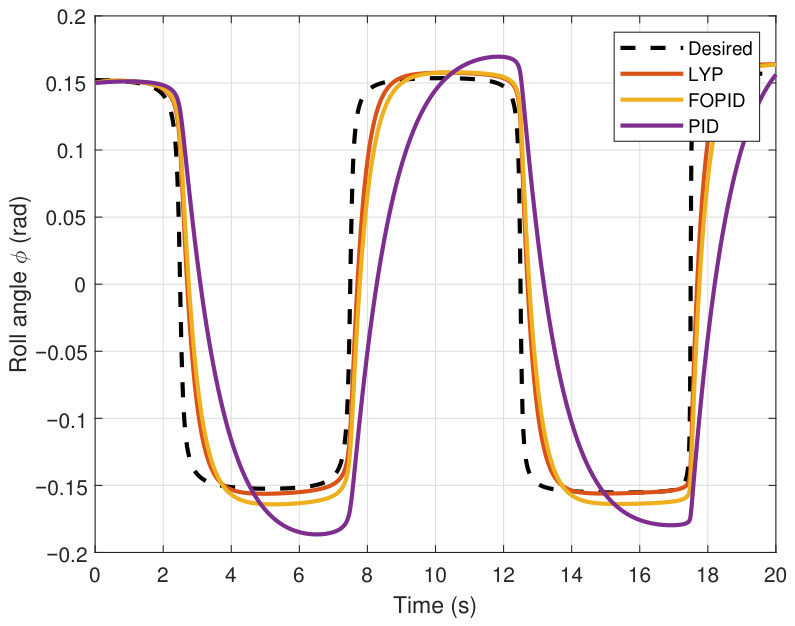
The roll angle result of tracking the figure-eight path.

**Figure 35:**
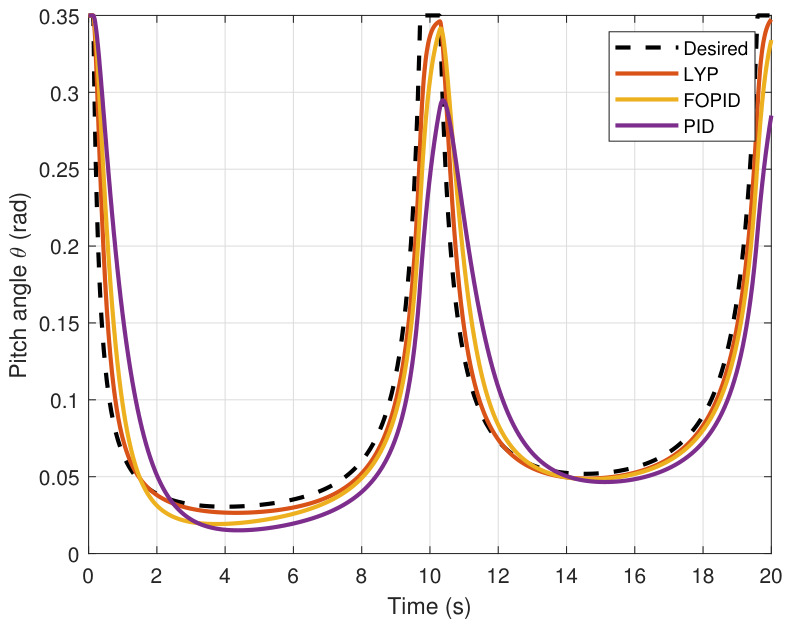
The pitch angle result of tracking the figure-eight path.

**Figure 36:**
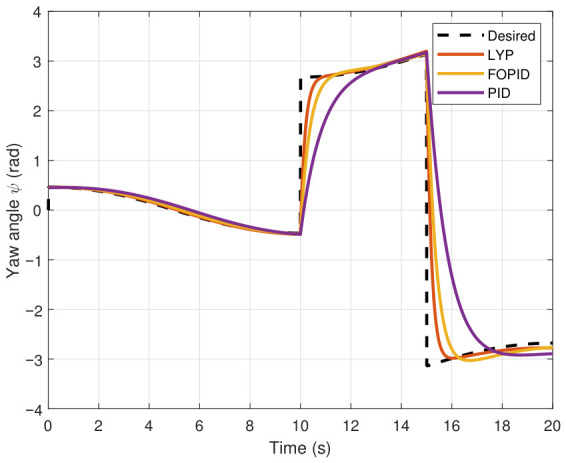
The yaw angle result of tracking the figure-eight path.

**Figure 37:**
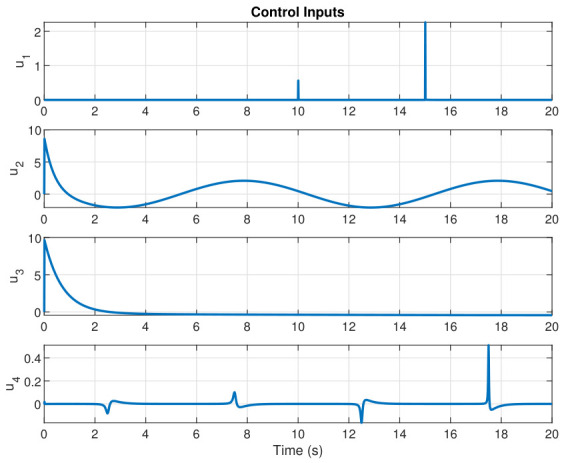
The control input for the figure-eight path.

Three controllers are evaluated in this section: PID, FOPID, and the proposed Lyapunov-based backstepping (LYP) controller. The tracking data are shown in Figure 31, Figure 32, Figure 33, Figure 34, Figure 35 and Figure 36, where the actual trajectory of position and altitude over a 20-s period is represented by a black dashed line. LYP is shown in red. FOPID is shown in yellow, and PID is shown in purple. Both LYP and FOPID offer strong tracking performance and stable quadrotor attitudes. Figure 37 shows the four control inputs. While $U_{1}$ and $U_{2}$ show greater amplitude changes, $U_{3}$ and $U_{4}$ stay smooth and rapidly stabilize after a brief transient period. The performance indices are summarized using the root mean square error (RMSE) in Table 6. The LYP controller features the best overall performance, despite the FOPID controller outperforming PID and achieving acceptable results.

## 9. Conclusions

The control of an underactuated quadrotor UAV with nonlinear dynamics and high sensitivity to disturbances and parameter uncertainty was examined in this study. A Lyapunov-based backstepping (LYP) controller with an inner–outer loop structure was developed to address the above difficulties and to provide accurate and stable trajectory tracking for both position and attitude control. The Newton–Euler equation provided the basis for the mathematical model of a six-degree-of-freedom quadrotor UAV. A proposed Lyapunov-based backstepping (LYP) controller, along with Particle Swarm Optimization (PSO), was designed and evaluated. PSO was used to optimize controller gains and improve performance while maintaining Lyapunov stability. LYP was compared with Fractional-Order PID (FOPID) and Proportional–Integral–Derivative (PID) controllers for controlling the UAV’s position and attitude. The controllers’ performance was evaluated in three scenarios: normal conditions, in the presence of external disturbance, and under strong parameter uncertainty. According to simulation results from a comparative analysis using time-domain performance indicators such as rising time, overshoot, settling time, and root mean square error (RMSE), the proposed new controller (LYP) demonstrated superior performance, with good disturbance rejection and parameter uncertainty control. The FOPID controller showed sufficient but not as good a performance as LYP. Both LYP and FOPID controllers demonstrated much better performance than the PID controller. Both controllers (LYP and FOPID) achieved satisfactory performance in reference trajectory tracking, while the proposed LYP controller was more robust to disturbance rejection and to parameter uncertainty correction. The most significant advances of this work are clearly indicated in the design of a Lyapunov-based backstepping controller with improved robustness. In addition, PSO is integrated for efficient and optimal gain adjustment. Finally, an extensive comparative study is conducted against PID and FOPID controllers under various challenging conditions. Overall, the results demonstrate that the Lyapunov-based backstepping technique is a superior and dependable control method for quadrotor UAV systems operating under uncertain parameters and unstable environments.

## Figures and Tables

**Table 1:** Parameters of the quadrotor (ASC-2500).

Variable	Value
m	0.13kg
$I=\mathrm{diag}(I_{x},I_{y},I_{z})$	[0.025, 0.023, 0.0374] kg·m^2^
*g*	$9.81\;{m/s}^{2}$
*L*	0.34m
*W*	0.36m
Camera HD	1080p

**Table 2:** A comparison of the controller’s response under normal conditions.

State	Controller	Rise Time (s)	Settling Time (s)	Overshoot (%)
X	PID	0.325	3.319	35.654
	FOPID	0.255	2.515	12.555
	LYP	0.250	3.192	6.468
Y	PID	0.386	6.402	31.054
	FOPID	0.205	1.938	10.079
	LYP	0.207	2.679	5.438
Z	PID	0.320	7.389	20.987
	FOPID	0.248	2.243	12.328
	LYP	0.230	3.902	6.286
Roll Angle	PID	0.644	5.345	26.270
	FOPID	0.318	3.332	11.705
	LYP	0.284	3.568	7.980
Pitch Angle	PID	0.621	5.345	29.544
	FOPID	0.285	2.302	13.906
	LYP	0.291	3.860	6.817
Yaw Angle	PID	0.493	4.184	29.356
	FOPID	0.319	3.332	11.705
	LYP	0.284	3.568	8.040

**Table 3:** A comparison of the controller’s response under disturbance.

State	Controller	Rise Time (s)	Settling Time (s)	Overshoot (%)
X	PID	0.365	14.344	42.881
	FOPID	0.262	12.870	18.440
	LYP	0.220	**12.616**	8.667
Y	PID	0.410	13.243	38.192
	FOPID	0.242	12.801	15.356
	LYP	0.182	12.709	7.258
Z	PID	0.339	12.981	28.052
	FOPID	0.273	12.841	30.813
	LYP	0.188	12.963	15.798
Roll Angle	PID	0.611	13.480	37.032
	FOPID	0.290	12.863	14.425
	LYP	0.314	12.807	9.525
Pitch Angle	PID	0.655	13.526	31.899
	FOPID	0.317	12.957	18.534
	LYP	0.291	12.758	9.970
Yaw Angle	PID	0.893	13.153	42.199
	FOPID	0.407	12.984	14.859
	LYP	0.299	12.743	9.710

**Table 4:** A comparison of the controller’s response with 50% parameter uncertainty.

State	Controller	Rise Time (s)	Settling Time (s)	Overshoot (%)
X	PID	0.304	5.132	54.885
	FOPID	0.239	1.854	15.615
	LYP	0.201	2.609	6.420
Y	PID	0.320	3.130	35.160
	FOPID	0.206	1.393	17.214
	LYP	0.217	2.871	5.428
Z	PID	0.306	3.166	28.352
	FOPID	0.217	1.699	14.202
	LYP	0.306	3.822	6.328
Roll Angle	PID	0.558	5.924	34.540
	FOPID	0.368	2.574	17.515
	LYP	0.287	3.592	7.857
Pitch Angle	PID	0.661	7.301	42.025
	FOPID	0.410	2.581	21.242
	LYP	0.316	3.628	6.993
Yaw Angle	PID	0.779	9.545	41.182
	FOPID	0.543	3.710	19.531
	LYP	0.287	3.592	8.037

**Table 5:** The performance on the circular path, as measured using the RMSE.

Controller	X-Position	Y-Position	Z-Position	Roll Angle	Pitch Angle	Yaw Angle
LYP	0.5090	0.5530	0.0014	0.0336	0.0001	0.0055
FOPID	0.9285	0.9416	0.0024	0.0845	0.0003	0.0193
PID	1.7602	2.4874	0.0122	0.3786	0.0006	0.4778

**Table 6:** The RMSE performance for the figure-eight path.

Controller	*x*-Position	*y*-Position	*z*-Position	Roll Angle	Pitch Angle	Yaw Angle
LYP	0.5506	0.1551	0.0003	0.1704	0.0013	0.2198
FOPID	0.8603	0.3112	0.0006	0.2417	0.0040	0.4120
PID	2.8716	0.9571	0.0010	0.8226	0.0099	0.9467

## Data Availability

The data supporting the findings of this study are available within the article. No additional datasets were generated or analyzed beyond those presented in this study.
